# Markers of oxidative stress in umbilical cord blood from G6PD deficient African newborns

**DOI:** 10.1371/journal.pone.0172980

**Published:** 2017-02-24

**Authors:** Paul S. Stadem, Megan V. Hilgers, Derrick Bengo, Sarah E. Cusick, Susan Ndidde, Tina M. Slusher, Troy C. Lund

**Affiliations:** 1 Mayo Clinic School of Medicine, Mayo Clinic, Rochester, Minnesota, United States of America; 2 Division of Global Health, Department of Pediatrics, University of Minnesota University, Minneapolis, Minnesota, United States of America; 3 Department of Hematology, Mulago Hospital, Kampala, Uganda; Laurentian University, CANADA

## Abstract

Glucose-6-phosphate dehydrogenase (G6PD) deficiency is an X-linked disorder that affects as many as 400 million people worldwide, making it the most common enzymatic defect. Subjects with G6PD deficiency are more likely to develop neonatal hyperbilirubinemia potentially leading to kernicterus and are at increased risk for acute hemolytic anemia when exposed to pro-oxidant compounds such as anti-malarial drugs. We collected umbilical cord blood from 300 males born in Uganda to assess for novel markers of systemic oxidative stress. We determined that 10.7% of the samples collected were G6PD A- deficient (G202A/A376G) and when these were compared with unaffected controls, there was significantly higher 8-hydroxy-2’-deoxyguanosine (8-OHdG) concentration, elevated ferritin, increased leukocyte count and higher small molecule antioxidant capacity. These data suggest increased baseline oxidative stress and an elevated antioxidant response in umbilical cord blood of patients with G6PD deficiency.

## Introduction

Glucose-6-phosphate dehydrogenase (G6PD) deficiency is an X-linked disorder that affects as many as 400 million people worldwide, making it the most common human enzymatic defect. [1] It is prevalent in Sub-Saharan Africa, where an estimated 10–25% of individuals are G6PD deficient. [2] In this region, the *G6PD A-* (G202A/A376G) mutation is most prevalent. [2] G6PD is a key enzyme in the oxidative pentose phosphate pathway that leads to the generation of nicotinamide adenine dinucleotide phosphate (NADP)H allowing the regeneration of reduced glutathione (GSH) from it’s oxidized form glutathione disulfide (GSSG). GSH is an important reducing agent to neutralize reactive oxygen species via reducing equivalent donation. Exposure of G6PD deficient individuals to pro-oxidants such as anti-malarial medications, fava beans or infections can induce acute hemolytic anemia and potentially progression to shock. [1, 3] Newborns with G6PD deficiency are more likely to develop hyperbilirubinemia and jaundice, although this often occurs in the absence of any known trigger or hemolysis. Neonates exposed to pro-oxidants such as mentholatum, naphthalene, and henna. [4–8] As well, many newborns develop severe hyperbilirubinemia in the absence of any identifiable trigger of hemolysis. Severe neonatal hyperbilirubinemia can lead to kernicterus with significant morbidity and mortality. [9–11]

Currently, there is little data on other biomarkers associated with G6PD deficiency data outside of bilirubin. Our goal was to explore potential markers of oxidative stress that are associated with G6PD deficiency at birth. Identification of such markers may help us better understand the pathophysiology of G6PD deficiency.

## Materials and methods

### Sample collection

This study and the use of umbilical cord blood (UCB) were approved by the Committees on the Use of Human Subjects in Research at the University of Minnesota and at Mulago Hospital in Uganda and conducted according to the principles expressed in the Declaration of Helsinki. Umbilical cord blood from 300 consecutive males born at Mulago National Referral Hospital in Kampala, Uganda was collected into EDTA blood tubes and samples were de-identified. Each sample was initially tested for G6PD deficiency using the Beutler florescent spot test and confirmed later by PCR using protocols previously described. [2, 12] A complete blood count (CBC) was obtained using a Nihon Kohden hematology analyzer. Samples were stored at -25 degrees Celsius for later testing.

### Assays for markers of oxidative stress

All assays were analyzed on a Tecan Sunrise microplate reader. The markers of inflammation/oxidative stress that we measured were as follows: 8-hydroxy-2’-deoxyguanosine (8-OHdG) (Trevigen, Gaithersburg, MD), protein carbonylation concentration (OxiSelect Protein Carbonyl ELISA Kit, Cell BioLabs), small molecule antioxidant capacity (Total Antioxidant Capacity Assay Kit, Abcam), catalase activity (StressXpress Colorimetric Activity Kit, StressMarq Biosciences Inc), and ferritin (Ramco Laboratories, Stafford, TX) according to the manufacturers’ protocol.

### Statistical methods

Means for the G6PD deficient and control groups were each calculated and compared with a two-tailed Student’s t-test. Pearson’s was used for correlation of plasma 8-OHdG levels and absolute lymphocyte counts. The raw data for this study is contained in S1 Table.

## Results and discussion

**Fig 1:**
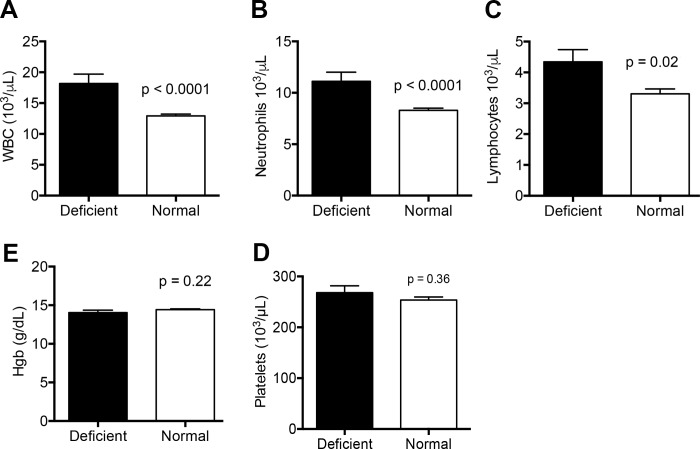
Complete blood count indices in G6PD deficient UCB from male neonates. (A-E) Total white blood cell (WBC), neutrophil, lymphocyte, platelet counts and hemoglobin were in G6PD deficient samples (n = 32) compared to normal controls (n = 240). Columns indicate the mean values and standard errors. P-values in two-way comparisons were derived from a Student’s t-test. Platelet values are from 23 G6PD deficient and 136 normal controls due to equipment malfunction. See also S1 Table.

We collected umbilical cord blood from 300 males born at Mulago Hospital in Kampala, Uganda and found 32 were G6PD A- deficient (10.7%) using the fluorescent spot test and PCR confirmation. This is consistent with previously reported rates. [2] We measured CBCs from each sample and found significant elevations in the total number of lymphocytes (4.3 x 10^3^/μL versus 3.3 x 10^3^μL, p = 0.02) and neutrophils (11.1 x 10^3^/μL versus 8.3 x 10^3^μL, p < 0.0001) associated with G6PD deficiency (Fig 1A–1C). Increased lymphocytes and neutrophils contributed to a significantly elevated white blood cell count in G6PD deficient neonates (18.2 x10^3^/μL versus 12.9 x10^3^/μL, p < 0.0001; Fig 1). While clinical conditions such as infection or prolonged labor could account for such differences, these were not noted and most neonates were discharged 24 hours after delivery without follow-up. Hemoglobin and platelet levels were similar between G6PD deficient and unaffected neonates (14.0 g/dL versus 14.4 g/dL, p = 0.22; 268 x 10^3^/μL versus 253 x 10^3^/μL, p = 0.36; Fig 1D and 1E), consistent with previous reports. [13, 14] It is generally accepted that unless persons with G6PD deficiency are exposed to a pro-oxidant trigger, hemolysis is not observed. [15] Furthermore, several reports find that there is not necessarily any identifiable trigger for neonatal hyperbilirubinemia in G6PD deficient infants, and assessments of hemolysis just after birth do not correlate with total serum bilirubin levels.[16, 17] This is not always true for those with severe forms of G6PD deficiency, who may have chronic levels of hemolysis. [15]

G6PD deficiency is the prototypical disease in which oxidative stress incites a hemolytic crisis. While there was no evidence of hemolysis in the UCB samples in this study, we were interested to learn if there were markers of oxidative stress present at a subclinical level. Other biomarker studies have shown that end-tidal carbon monoxide and blood carboxyhemoglobin levels are elevated in newborns with G6PD deficiency, even in the absence of hemolysis. [18, 19] We investigated several known markers of oxidative stress and inflammation and found significant variation between G6PD deficient samples and the control group.

**Fig 2:**
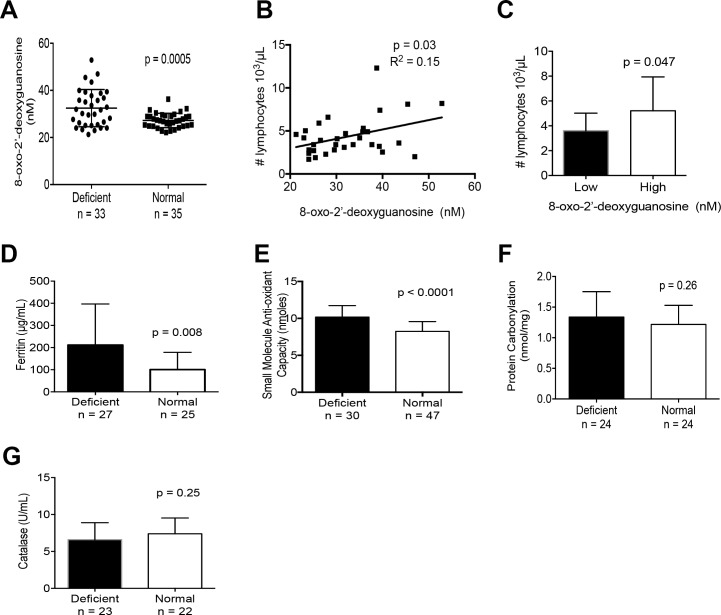
Markers of oxidative stress/inflammation in G6PD deficient UCB from male neonates. (A) Levels of plasma 8-OHdG, n = 32 G6PD deficient, n = 35 Normal samples. (B) Pearson’s correlation analysis of plasma 8-OHdG levels and absolute lymphocyte counts. (C) Comparison of G6PD deficient UCB lymphocyte counts between high and low 8-OHdG levels determine by the median 8-OHdG value (31.9 nM). (D) Plasma ferritin concentration in UCB. (E) Plasma small molecule antioxidant capacity expressed in equivalents of Trolox. (F) Plasma protein carbonylation as nmol/mg. (G) Plasma catalase activity in UCB. See also S1 Table. Columns indicate the mean values and standard deviation. P-values in two-way comparisons were derived from a Student’s t-test.

We quantified plasma 8-hydroxy-2’-deoxyguanosine (8-OHdG), an oxidized derivative of deoxyguanosine and commonly used as a nonspecific measure of oxidative stress. [20–22] The mean plasma 8-OHdG concentration was significantly elevated at 32.5 ng/mL in G6PD deficient samples compared to 27.2 ng/mL in controls (p = 0.0005; Fig 2A). We also found that higher levels of 8-OHdG correlated with elevated leukocytes in G6PD deficient neonates (Fig 1B and 1C). This observation suggests that 8-OHdG is linked to an inflammatory response in G6PD deficient neonates. In current literature, 8-OHdG has been interrelated to other measures of inflammation such as ferritin. [22, 23] Our study did also show significantly higher ferritin levels in UCB from G6PD deficient neonates (211.6 μg/mL vs 100.5 μg/mL, p = 0.008; Fig 2D), further supporting the hypothesis of a subclinical inflammatory response.

By definition, persons with G6PD deficiency have erythrocytes that are much less capable to regenerate NADPH after it is oxidized to NADP leading to reduced anti-oxidant capacity. We performed a global assessment of the anti-oxidant capacity in plasma by measuring the amount of Cu^2+^ converted to Cu^+^ standardized against Trolox, an analog of Vitamin E. Interestingly, we found that the mean small molecule antioxidant capacity in G6PD deficient samples was elevated at 5.10 nmol/μl compared to 4.12 nmol/μl in control samples (p < 0.0001; Fig 2E). This data suggests that there may be a biologic response to the abnormally low levels of G6PD activity that includes increasing plasma levels of small molecule antioxidants such as ascorbate, uric acid and vitamin E in a compensatory effort.

Protein carbonylation involves the carbonyl modification of amino acids that occurs during free radical generation. [24, 25] We did not find a significant difference in carbonylation between G6PD deficient and control samples (p = 0.26; Fig 2F), thus we speculate that any preceding oxidative challenge may have been too brief to allow this modification to occur in UCB. Catalase has been previously reported to be reduced in Mediterranean men after fava bean ingestion [26], though we found no significant difference between G6PD deficient UCB samples and controls (p = 0.25; Fig 2G. The lack of G6PD deficiency’s effect on catalase activity has also been shown in a murine model of G6PD deficiency. [27]

This novel study in Sub-Saharan Africa shows the prevalence of G6PD deficiency in newborn males in Kampala, Uganda to be 10.7%. Our data suggest increased baseline oxidative stress and an elevated antioxidant response in UCB of patients with G6PD deficiency. Longitudinal follow-up was not a part of this study, however our results are the first to specify significant markers of oxidative stress in UCB of African newborns with G6PD deficiency. Further inquiry into the correlation between these markers and the development of clinically significant disease, such as hyperbilirubinemia, hemolysis, and the development of kernicterus, may allow us to better understand the pathophysiology of G6PD deficiency in the newborn.

## Supporting information

S1 Table Raw data supporting the figures. (XLSX)
